# Endovenous laser ablation therapy in children: applications and outcomes

**DOI:** 10.1007/s00247-017-3863-4

**Published:** 2017-05-18

**Authors:** Premal A. Patel, Alex M. Barnacle, Sam Stuart, Joao G. Amaral, Philip R. John

**Affiliations:** 1 Image Guided Therapy, Department of Diagnostic Imaging, The Hospital for Sick Children, University of Toronto, Toronto, ON Canada; 2 Interventional Radiology, Department of Radiology, Great Ormond Street Hospital for Children NHS Foundation Trust, Great Ormond Street, London, WC1N 3JH UK; 3 Translational Imaging Group, Centre for Medical Image Computing, Department of Medical Physics and Biomedical Engineering, University College London, London, UK

**Keywords:** Children, Endovenous laser ablation, Klippel–Trenaunay syndrome, Varicose veins, Venous malformation

## Abstract

**Background:**

Endovenous laser ablation is well recognized as the first-line treatment for superficial venous reflux with varicose veins in adults. It is not widely reported and is not an established practice in pediatric patients.

**Objective:**

To illustrate a variety of pediatric venous conditions in which endovenous laser ablation can be utilized and to demonstrate its feasibility and safety in children.

**Materials and methods:**

We conducted a retrospective review of endovenous laser ablation procedures performed between January 2007 and July 2014 at two large pediatric institutions.

**Results:**

We included 35 patients (17 males) who underwent endovenous laser ablation to 43 veins. Median age at first treatment was 14 years (range: 3–18 years). Median weight was 56 kg (range: 19–97 kg). Underlying diagnoses were common venous malformation (15), Klippel–Trenaunay syndrome (8), superficial venous reflux with varicose veins (5), verrucous hemangioma-related phlebectasia (4), venous varix (2) and arteriovenous fistula (1). The most common aim of treatment was to facilitate sclerotherapy. Thirty-four patients had treatment in the lower limbs and one patient in an upper limb. Ten of the veins treated with endovenous laser ablation had an additional procedure performed to close the vein. Complications attributable to endovenous laser ablation occurred in two patients (6%). One patient experienced post-procedural pain and one patient developed a temporary sensory nerve injury. Median clinical follow-up was 13 months (range: 28 days–5.7 years). The aim of the treatment was achieved in 29 of the 35 (83%) patients.

**Conclusion:**

Endovenous laser ablation is technically feasible and safe in children. It can be used in the management of a range of pediatric venous diseases with good outcomes.

## Introduction

Since the first use of endoluminal laser energy was reported in 1999 [1], its clinical use has grown and it has been shown in adults to be both safe and effective for the treatment of varicose veins related to superficial venous incompetence [2, 3]. Endovenous laser ablation is now considered a first-line treatment option for varicose veins related to superficial venous reflux [4].

The use of endovenous laser ablation is not widely reported and is not an established practice in pediatrics. There are, however, several venous disorders in children in which endovenous laser ablation can play a useful role. We report our experience utilizing endovenous laser ablation in children of various ages to treat a spectrum of venous disorders including some that are outside the indication for which the technique was originally designed. We demonstrate the feasibility of endovenous laser ablation in children, illustrate a variety of pediatric venous diseases in which endovenous laser ablation can be successfully utilized, and report on the safety of the technique.

## Materials and methods

### Patients

We obtained institutional research ethics board approval for this two-center retrospective observational study. We collected data from two large pediatric institutions with both pediatric vascular anomaly services and well-established pediatric interventional radiology programs (The Hospital for Sick Children, Toronto, Canada, and Great Ormond Street Hospital for Children NHS Foundation Trust, London, UK). We included all consecutive patients 0–18 years of age who underwent endovenous laser ablation between January 2007 and July 2014. There were no exclusion criteria. We identified these patients by searching each institution’s interventional radiology database for the procedure “endovenous laser ablation.” Data sources included the institutional picture archiving and communication systems, prospectively collected dedicated interventional radiology databases and paper or electronic patient charts.

Demographics recorded included age, weight at the time of endovenous laser ablation and gender. We also recorded pre-procedural diagnosis, symptoms and imaging findings. Procedural data included aim of treatment, number and nature of veins treated, endovenous laser ablation technique, additional endovascular procedures performed at the same time as endovenous laser ablation and recovery time. Post-procedural data collected included postoperative imaging, complications, outcome and length of follow-up. Complications were graded according the Society of Interventional Radiology classification of complications [5].

### Endovenous laser ablation technique

Informed consent for endovenous laser ablation with or without additional endovascular procedures was obtained prior to all procedures. The procedures were performed under sterile conditions in an angiography suite. Procedures were performed under general anesthesia with continuous monitoring of vital functions. The target vein was accessed percutaneously by using a micropuncture technique with US guidance. A 4-French laser sheath was advanced over a 0.035-in. wire to the intended start point of ablation. The wire was exchanged for the laser fiber, ensuring the laser fiber tip remained covered by the sheath. Tumescent fluid was injected around the entire length of the target vein. The tumescent fluid used was 0.9% sodium chloride solution in 37 procedures, and lactated Ringer solution in 4 procedures. Lidocaine was added to the fluid in six procedures early in the series; no local anesthetic was used for the other procedures. Because of target vein length, four veins required two access sites and one vein required three access sites. Where multiple access sites were used, access was gained at all sites before administration of tumescent fluid. An 810-nm Diode laser with 600-μm bare-tip fiber was used. The endovenous laser ablation systems were either Vari-Lase (Aquilant Interventional, Hampshire, UK) or Diomed EVLT (AngioDynamics, New York, NY). The power used was 10–14 watts in continuous mode with a pullback rate of 1–2 mm/s. Immediately following the procedure, either 20–30 mmHg or 30–40 mmHg compression garments were applied and worn for 6–12 weeks. Eight patients received prophylactic peri-procedural anticoagulation; five of these patients were known to be prothrombotic. Endovenous laser ablation was preceded by venography or venous outflow embolization and followed by sclerotherapy in select cases.

## Results

Thirty-five patients (17 males) underwent a total of 41 endovenous laser ablation procedures to treat 43 veins (more than one vein was ablated at some procedures; Table 1). Median age at first treatment was 14 years (range: 3–18 years). Median weight at first procedure was 56.4 kg (range: 19.1–96.9 kg). The patients were divided into and analyzed in four groups according to the underlying diagnosis [6]: common venous malformation in 15 patients, Klippel–Trenaunay syndrome in 8 patients, superficial venous reflux with varicose veins in 5 patients, and a group for other miscellaneous vascular disorders comprising verrucous hemangioma-related phlebectasia in 4 patients, venous varix in 2 patients and arteriovenous fistula in 1 patient. Endovenous laser ablation was performed in the lower limbs in 34 patients (19 right, 14 left, 1 bilateral) and in the right upper limb in 1 patient. Three patients required more than one endovenous laser ablation procedure to ablate the target vein.

**Table 1:** Summary of all patients treated with endovenous laser ablation by group

Patient group	Number of patients	Age (y), median (range)/gender	Clinical problem / reason to treat	Numbers of veins treated	Target vein(s)	Number of endovenous laser ablation procedures	Complications of endovenous laser ablation	Clinical outcome	Imaging outcome	Average length of follow-up (days)
Venous malformations	15	14 (7–17) 7 M, 8 F	Control outflow from venous malformation for sclerotherapy (12) Prevent venous thrombosis (2) Prevent thrombophlebitis (1)	18	Greater saphenous vein (8) Embryonic vein (5)^a^ Immediate lesion draining vein (2) Dysplastic vein (2)^b^ Short saphenous vein (1)	18	Temporary sensory nerve injury (1)	Sclerotherapy successfully performed (10) Symptoms resolved (3) Aim of treatment not achieved (2)	Closed (5) Partially closed (7) Patent (3)	788
Klippel–Trenaunay syndrome	8	16.5 (7–18) 4 M, 4 F	Pain and swelling (4) Pain (1) Cosmetic (1) Control outflow from venous malformation for sclerotherapy (1)	9	Greater saphenous vein (2) Embryonic vein (7)	9	Pain (1)	Sclerotherapy successfully performed (1) Symptoms resolved (6), Improved but persistent pain (1)	Closed (5) Partially closed (1) Patent (1) No imaging follow-up (1)	213
Varicose veins	5	15 (13–16) 3 M, 2 F	Pain and swelling (1) Pain (3) Cosmetic (1)	7	Greater saphenous vein (6) Accessory greater saphenous vein (1)	6	None	Symptoms resolved (3) Persistent pain (1) Persistent varicosities (1)	Closed (3) Partially closed (1) No imaging follow-up (1)	142
Miscellaneous pathology	7	9 (3–16) 3 M, 4 F	Facilitate arteriovenous fistula closure (1) Reduce pain & swelling secondary to large varix (2) Reduce pain and swelling secondary to verrucous hemangioma-related phlebectasia (3) Facilitate surgery for verrucous hemangioma-related phlebectasia (1)	9	Greater saphenous vein (2) Embryonic vein (3)^a^ Immediate lesion draining vein (1) Dysplastic vein (3)^b^	8	None	arteriovenous fistula successfully closed (1), Surgery successfully performed (1), Symptoms resolved (4) Symptoms persisted (1)	Closed (2) Partially closed (4) No imaging follow-up (2)	1,040

*F* female, *M* male, *y* years

^a^Embryonic veins unrelated to Klippel–Trenaunay syndrome

^b^Non-embryonic dysplastic veins

In addition to endovenous laser ablation, adjunctive closure techniques were used for 10 veins in 10 patients. Six of these veins were embryonic veins; all were occluded with an Amplatzer Vascular Plug (St. Jude Medical Inc., St. Paul, MN) to close the intrafascial deep cephalic component, and two of these had additional sclerotherapy with sodium tetradacyl sulfate 3% foam. Three veins were greater saphenous vein and one was a non-embryonal dysplastic vein; these all had sclerotherapy with sodium tetradacyl sulfate foam. The choice of adjunctive closure method was based on the intervening radiologist’s preference.

Early in the series, eight patients were electively admitted overnight post-procedure; in one of these cases, the admission was extended to two nights for management of pain. Thirty-three procedures were conducted in outpatients. The overall aim of the treatment was achieved in 29 of the 35 (83%, 95% confidence interval [CI] 70–95%) patients.

### Patients with common venous malformations

**Fig. 1:**
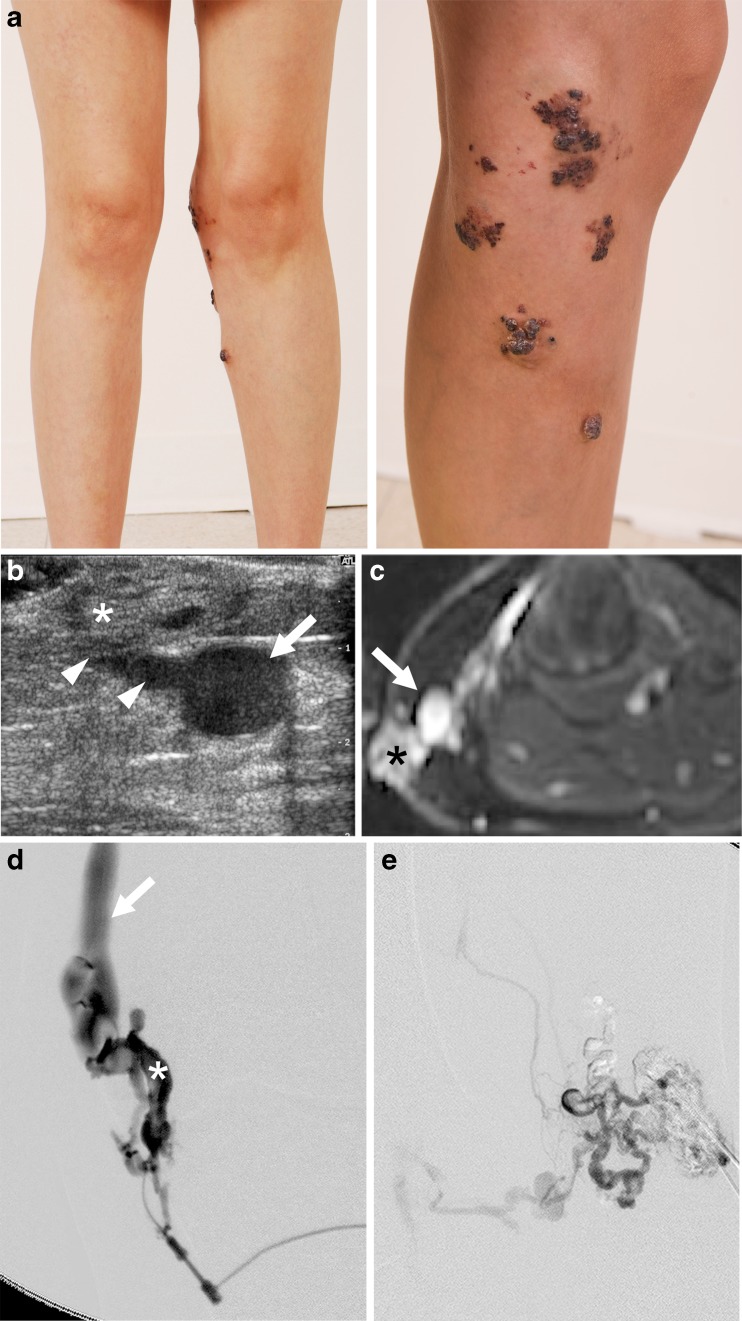
Multifocal venous malformations in a 16-year-old girl. **a** Clinical photographs demonstrate multifocal venous malformation located in skin and subcutaneous tissue at the knee. Venous malformations were also located in intramuscular tissues of the upper and lower thigh (not shown). **b**, **c** Ultrasound (**b**) and axial contrast-enhanced T1-weighted fat-saturated MRI (**c**) of subcutaneous soft tissues on the medial aspect of the knee show an ectatic greater saphenous vein *(arrow)* with connecting draining veins *(arrowheads)* from the subcutaneous component *(asterisk)* of the skin venous malformations. **d** Direct puncture venogram of subcutaneous venous malformation *(asterisk)* shows direct communication with the greater saphenous vein *(arrow)*. Endovenous laser ablation of the greater saphenous vein was performed. **e** Repeat venogram of subcutaneous venous malformation following endovenous laser ablation of the greater saphenous vein shows reduced communication with the greater saphenous vein, which allowed for sclerotherapy of the venous malformation. Following endovenous laser ablation and sclerotherapy, pain and medial knee swelling improved and selected skin venous malformations became flatter and paler

Eighteen lower-limb veins were ablated in 15 patients with venous malformations (right in 8 patients, left in 7 patients) (Table 1). In 12 patients, the aim of endovenous laser ablation was to close the venous outflow from the lesion to enable safe injection of sclerosant into the venous malformation (Fig. 1). In two patients, endovenous laser ablation was performed in the greater saphenous vein to manage venous thrombosis — in one to prevent recurrence of distal massive thrombus, which had formed at a previous sclerotherapy treatment, and in the other to prevent thrombus propagation and pain after thrombus developed following sclerotherapy. In one patient, endovenous laser ablation of the short saphenous vein was performed to reduce symptoms of recurrent thrombophlebitis. One vein required three separate endovenous laser ablation procedures and one vein required two procedures to achieve closure. Six patients had additional procedures to close the ablated vein at the time of endovenous laser ablation: four had sclerotherapy post-ablation and two underwent pre-ablation endovascular occlusion with an Amplatzer Vascular Plug IV. Three patients were temporarily anticoagulated pre- and post-endovenous laser ablation: one had underlying coagulopathy and two had a history of recurrent superficial venous thrombosis.

The aim of the endovenous laser ablation was achieved in 13 of the 15 patients (87%). In both patients in whom the aim of endovenous laser ablation was not achieved, it had been performed to facilitate sclerotherapy. Neither patient went on to have sclerotherapy: one had persistent symptoms and a patent vein on ultrasound at 43 days post ablation; the other underwent an attempt to perform sclerotherapy but had no accessible channels and symptoms persisted. Despite achievement of the clinical aim of endovenous laser ablation in 13 patients, long-term vein closure, assessed at follow-up imaging, was not always achieved. There was no imaging follow-up in one patient after two endovenous laser ablations to a single vein.

### Patients with Klippel–Trenaunay syndrome

Eight patients with Klippel–Trenaunay syndrome (Table 1) were treated with the aim of improvement of pain or extremity swelling in five, cosmetic improvement of varicosities in two and to facilitate sclerotherapy by closing draining veins in one. Prior to endovenous laser ablation, all patients underwent venography to prove patency of the deep venous system and demonstrate anatomy of the target veins.

**Fig. 2:**
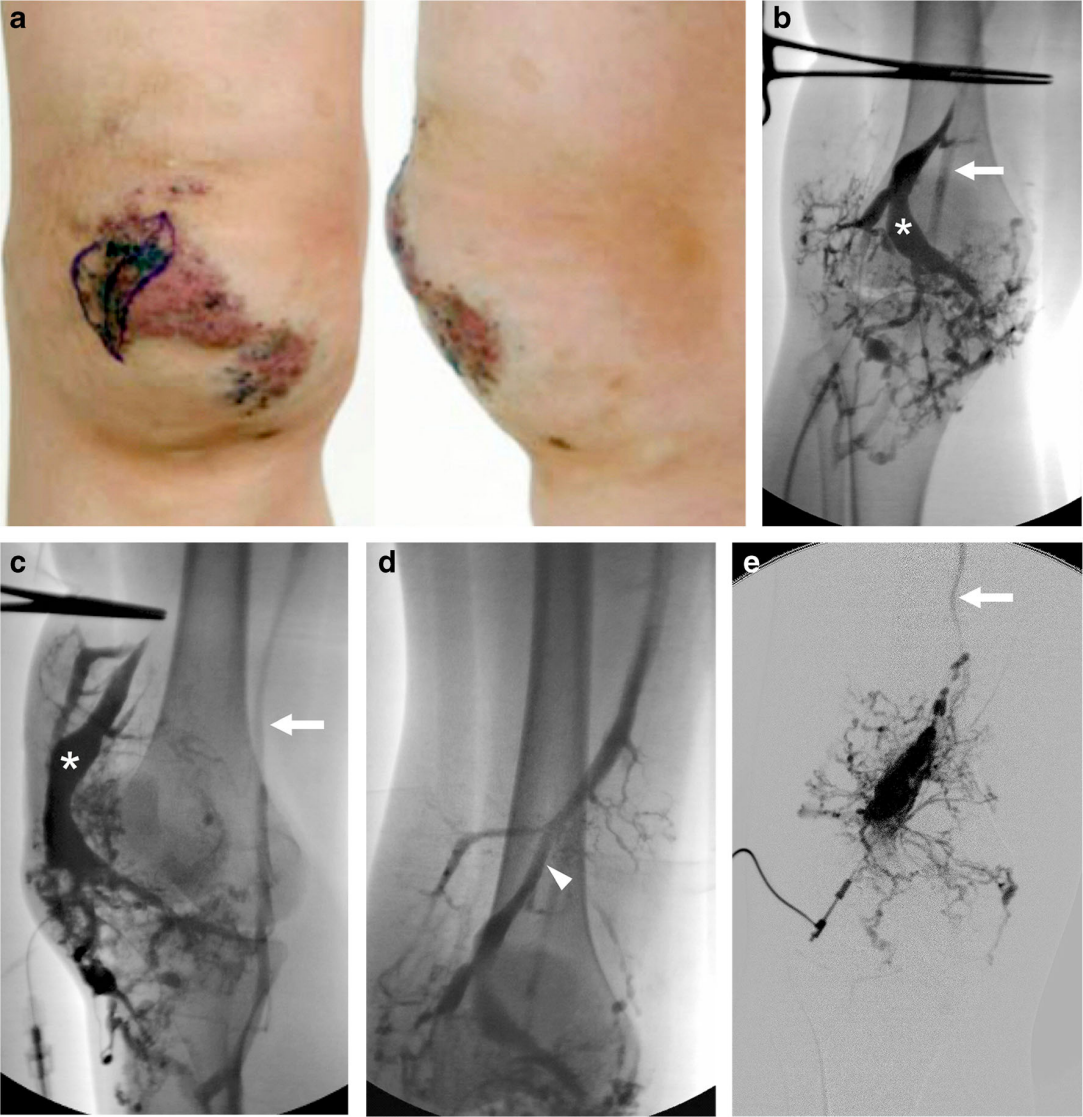
Klippel–Trenaunay syndrome in a 15-year-old girl. **a** Clinical photographs demonstrate knee swelling (from subcutaneous venous malformation). Ink mark on the cutaneous “geographic capillary malformation” was done by the surgeon prior to local excision of lymphatic vesicles on capillary malformation. **b–e** Direct puncture venograms of subcutaneous venous malformation on anterior knee. A large central vein *(asterisks)* is seen draining into the popliteal vein *(arrows)* when tourniquet is applied across lower thigh on both the frontal (**b**) and oblique (**c**) projections. With thigh tourniquet released **(d)** prompt drainage is seen superiorly from venous malformation into the embryonic vein in the anterior thigh *(arrowhead)*. Following endovenous laser ablation of the embryonic vein, the venous outflow from the venous malformation into the embryonic vein is reduced, allowing for more effective sclerotherapy of the venous malformation **(e)**

One patient undergoing endovenous laser ablation to an embryonic vein required two pullbacks of the laser to close the vein adequately (Fig. 2). One patient required repeat endovenous laser ablation of the greater saphenous vein because the lower end of the greater saphenous vein initially remained patent. Three patients had endovascular placement of an Amplatzer Vascular Plug II to close the intra-fascial deep cephalic component of the embryonic vein and prevent subsequent thromboembolism to the central veins. Two of these also underwent sclerotherapy with sodium tetradecyl sulfate (STS) foam (Sotradecol; Bioniche Pharma USA, Lake Forest, IL) at the time of endovenous laser ablation. Sclerotherapy with STS foam was undertaken in one patient 49 days post endovenous laser ablation to treat residual patency of the treated vein. Four patients received prophylactic peri-procedural anticoagulation. One patient had an inferior vena cava (IVC) filter placed at the same procedure as the endovenous laser ablation.

The aim of endovenous laser ablation was achieved in seven of the eight patients (88%) and partially achieved in one patient, who showed some improvement in pain and swelling with residual patency of the treated vein on follow-up imaging.

### Patients with superficial venous reflux with varicose veins

Five patients were treated for varicose veins (Table 1). Prior to endovenous laser ablation treatment, all patients had Duplex ultrasonography demonstrating superficial venous reflux. A total of seven greater saphenous veins were ablated. Endovenous laser ablation was commenced 2 cm below the sapheno-femoral junction in keeping with adult treatment recommendations [7]. In one patient endovenous laser ablation was done bilaterally; this patient had three veins (bilateral greater saphenous and an accessory greater saphenous vein) ablated over two endovenous laser ablation procedures. No adjunctive therapy was used at the time of treatment and none of these patients required anticoagulation.

The aim of the endovenous laser ablation was achieved in three of the five patients (60%). In the two patients where symptom improvement was not achieved, however, follow-up imaging did show vein closure. In one of these patients there was partial reduction in varicosities and in the other there was no improvement to the varicosity-related pain.

### Patients with miscellaneous anomalies

**Fig. 3:**
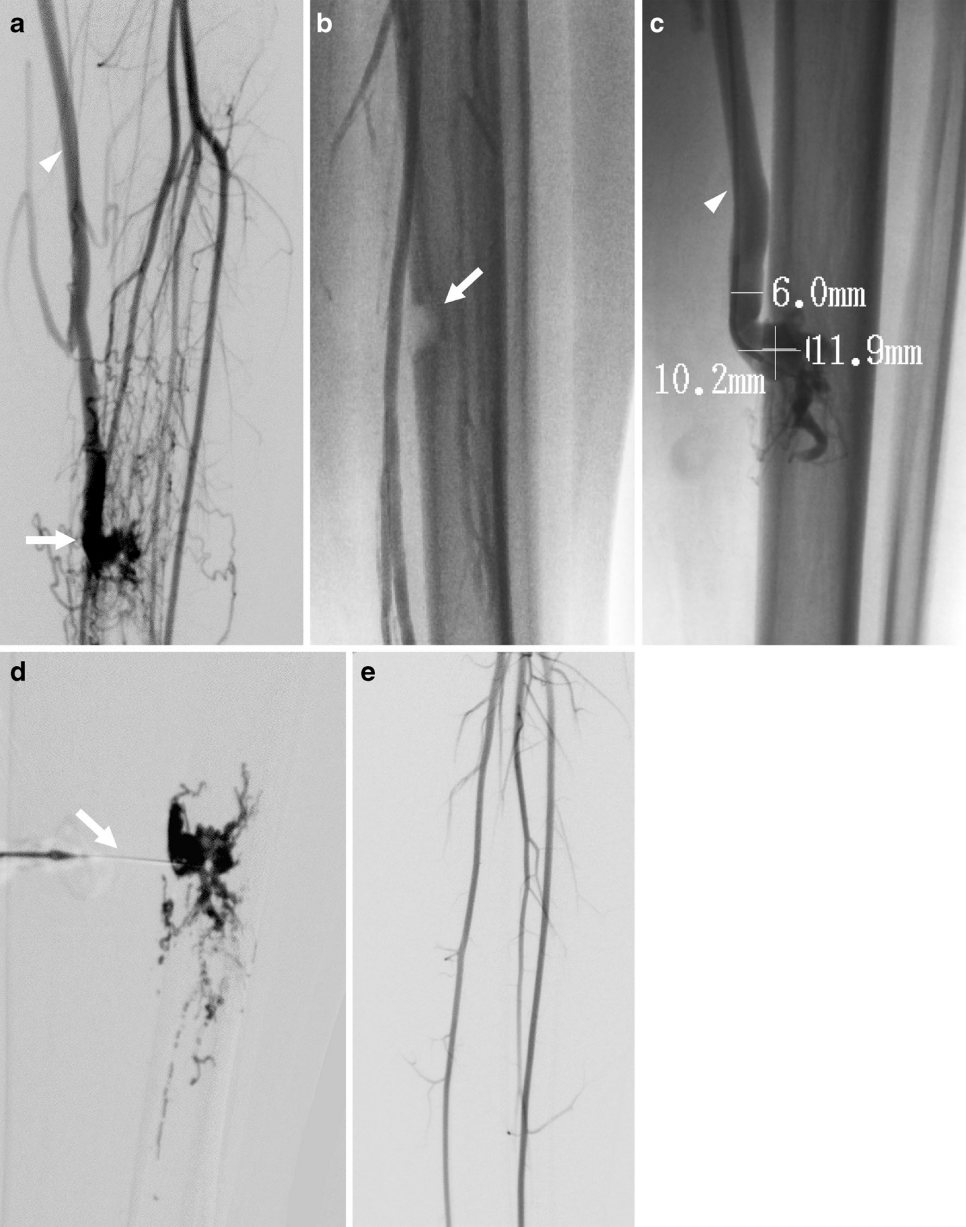
Arteriovenous fistula in a 16-year-old boy. **a** Arteriogram confirms arteriovenous fistula *(arrow)* supplied by tibial arteries with drainage into the greater saphenous vein *(arrowhead)*. **b** A bony defect in the tibia *(arrow)* caused by the arteriovenous fistula. **c** Direct venography of greater saphenous vein shows venous end of the arteriovenous fistula (at the tibial bony defect) draining into the greater saphenous vein *(arrowhead)*. Measurements of bony defect and vein were performed and endovenous laser ablation was then undertaken to occlude the greater saphenous vein. **d** Contrast agent injection following direct needle puncture of the venous end of the arteriovenous fistula *(arrow)* immediately after endovenous laser ablation was followed by embolization with glue. **e** Repeat arteriogram (immediately after endovenous laser ablation and glue embolization) confirms arteriovenous fistula closure

There were seven other patients, with a range of underlying diagnoses treated, with nine veins treated with endovenous laser ablation in eight procedures (Table 1). In one patient, endovenous laser ablation was used to close the draining vein of an arteriovenous fistula to facilitate subsequent direct glue embolization of the fistula (Fig. 3). Two patients were treated to reduce pain and swelling and to facilitate sclerotherapy of large superficial varicosities. Four patients with verrucous hemangiomas were treated, with the aim to facilitate sclerotherapy of verrucous hemangioma associated phlebectasia in two, facilitate surgical excision of the lesion in one, provide cosmetic improvement in one and reduce symptoms of pain and swelling in three (there was more than one indication in some patients). One patient with verrucous-hemangioma-related phlebectasia had two procedures: the first was to close an associated embryonic vein; however the patient remained symptomatic and was found to have a new enlarged embryonic vein, so this was closed at the second procedure. None of the treated veins required adjunctive closure techniques at the time of the endovenous laser ablation. Only the patient with the arteriovenous fistula received intra-procedural anticoagulation.

The aim of the endovenous laser ablation was achieved in six of the seven patients (86%). The patient in whom the aim of treatment was not achieved had a non-embryonic dysplastic vein partially closed at follow-up imaging; however symptoms of pain and swelling only resolved after the vein was ligated 199 days post endovenous laser ablation. In the six patients in whom the aim of treatment was achieved at follow-up, the treated veins were closed in two patients and partially closed in the remainder.

### Complications

There were six complications following five procedures in five patients. Of these, two were directly attributable to the endovenous laser ablation procedure (6%, 95% confidence interval [CI] 0–13%). One was an episode of localized pain that resulted in extension of the patient’s hospital stay by 1 night (Society of Interventional Radiology category B, i.e. requiring nominal therapy with no consequence; Table 1). The other was a temporary cutaneous sensory nerve injury (Society of Interventional Radiology category A, i.e. requiring no therapy and with no consequence). This presented as sensory blunting noted at the first clinical follow-up 47 days post endovenous laser ablation to facilitate sclerotherapy in a patient with a venous malformation and subsequently resolved. The remaining complications were clinically determined to be unrelated directly to the endovenous laser ablation procedure and included: one temporary sensory nerve injury (Society of Interventional Radiology category A), which presented as altered skin sensation in distribution of sclerotherapy treatment, not endovenous laser ablation treatment; one 1-cm^2^ area of ulceration in the popliteal fossa following endovenous laser ablation of an embryonic vein (Society of Interventional Radiology category B), which based on the location was thought to be caused by reflux of STS; one IVC perforation by the strut of an IVC filter placed at the same time as the endovenous laser ablation treatment (Society of Interventional Radiology category C, i.e. requiring therapy or minor hospitalization <48 h); and one episode of musculoskeletal chest pain (Society of Interventional Radiology category B), which presented to the emergency department and required a workup including a negative CT pulmonary angiogram.

## Discussion

Endovenous laser ablation delivers thermal energy from a point source at the tip of the laser fiber in the lumen of the vein, causing vessel wall heating. Several mechanisms result in an increase in vein wall temperature, including the direct contact of the fiber tip, the optical-thermal interaction of laser light and surrounding tissues, the heat flow from carbonized blood around the fiber tip and the heat transfer from steam bubbles produced during endovenous laser ablation [8]. This results in collagen contraction and denudement of the endothelium, stimulating vein wall thickening with eventual luminal contraction and vein fibrosis. The laser energy is distributed along the length of the vein to be treated by retracting the laser fiber along the vein. This results in non-thrombotic vessel occlusion [3]. It is now understood that the efficacy of endovenous laser ablation is influenced less by the target chromophore (hemoglobin) and the wavelength of the laser light than was previously thought. Although laser energy at 810 nm wavelength, delivered via a 600 μm laser fiber, is widely used in clinical practice in adults, it is recognized that many wavelengths between 810 nm and 1,470 nm are effective in vein obliteration [9]. The efficacy of endovenous laser ablation is determined by several factors including the laser power (i.e. the fluence rate and not the laser energy), the pullback velocity and the vein diameter [9].

The literature to date regarding endovenous laser ablation has focused on its applications in adults, in whom endovenous laser ablation has been demonstrated to be effective and safe in the treatment of greater saphenous vein reflux with a long-term occlusion rate of 93% [3]. Clinical outcomes from endovenous laser ablation and surgical treatment for varicose veins related to sapheno-femoral reflux are in equipoise [2, 10, 11], and endovenous laser ablation has become the first-line treatment option for varicose veins [4].

There is a paucity of reports of the use of endovenous laser ablation in children [12, 13]. Interstitial laser ablation for vascular malformations using a neodymium:yttrium aluminum garnet laser was reported as early as 1986 [14]. Subsequently a cohort of six children with venous malformations was treated using an intralesional diode laser with good outcomes and no major complications [12]. More recently the successful use of endovenous laser ablation with a diode laser was reported in treating the embryonic marginal venous system in four children weighing less than 20 kg with Klippel–Trenaunay syndrome [13]. Our study combines data from two major pediatric tertiary referral hospitals and includes 35 patients, with the aim of the endovenous laser ablation being achieved in 29 (83%). Although to the best of our knowledge this is the largest published cohort of children undergoing endovenous laser ablation, in our series the 95% confidence interval is wide, ranging 70–95%.

Our results highlight differences that need to be taken into consideration when undertaking endovenous laser ablation in children as a result of both the variable venous diseases encountered and the specific requirements of pediatrics. For example, we found that endovenous laser ablation can be a useful treatment adjunct in patients undergoing intralesional sclerotherapy to treat soft-tissue venous malformations where the outflow control from the venous malformation cannot be achieved by simple measures such as an external tourniquet. In such cases endovenous laser ablation, by isolating the venous malformation from its venous outflow, allows for more effective intralesional sclerotherapy, as in 14 of our patients. Another example included in the cohort is a patient in whom endovenous laser ablation was used for closure of the venous outflow from an arteriovenous fistula. Closure of the vein allowed for subsequent glue embolization of the fistula. This novel application of endovenous laser ablation highlights the versatility of the treatment modality; however based upon a single patient we cannot comment on how successful or useful endovenous laser ablation may be for other such cases.

Children with Klippel–Trenaunay syndrome are a distinct subgroup because of their variable venous anatomy and predisposition to venothromboembolic events [15–17]. In these children there is typically a coexistent prominent marginal venous system and a deep venous system. When undertaking endovenous laser ablation of an extremity embryonic marginal vein, venographic assessment of both the deep venous system and the embryonic veins should be undertaken prior to endovenous laser ablation. Establishing patency and continuity of the deep venous system is mandatory before treatment of the embryonic vein is undertaken [16, 18]. In all our patients with Klippel–Trenaunay syndrome, pre-endovenous laser ablation venography was performed using a technique similar to that described by Alomari [16]. The embryonic marginal venous system is located within the lateral aspect of the extremity and is usually composed of valveless superficial sometimes thick-walled ectatic veins, which predispose to venous stasis and thromboembolism, particularly in the post-procedural period [16, 17]. Therefore these patients should have their coagulation parameters checked prior to endovenous laser ablation. Peri-procedural anticoagulation, under the supervision of a hematologist, should be considered because of the higher risk of venous thromboembolism in this population, particularly when procedures are expected to be complex and lengthy. We placed one IVC filter prior to endovenous laser ablation in one patient because of the higher risk of thromboembolism in that patient.

Planning the site for venous access is important when undertaking endovenous laser ablation in children because of the variable nature of the venous diseases encountered in pediatrics. For endovenous laser ablation treatment for varicose veins related to sapheno-femoral reflux, the preferred point of the greater saphenous vein puncture is just below the knee, as in adults, with endovenous laser ablation commencing 2 cm below the sapheno-femoral junction [7]. When ablating long or tortuous vein segments, as in five of our endovenous laser ablation treated veins, more than one venous entry site might be required. Vein tortuosity is considered by some interventional radiologists to be a contraindication to endovenous laser ablation [19]. This is because the relatively stiff laser fiber might not track well along the tortuous veins. However this was not found to be to be a problem in any of the patients in this cohort. Atasoy [19] reported 98% technical success when performing endovenous laser ablation in very large and tortuous great saphenous veins in adults. The author achieved this by using US-guided catheterization with support from the straight catheter of the laser and by using multiple access points when the former failed. This was carried out to avoid navigating tortuous vessels with angled-tip catheters, which can be time-consuming and risky for vasoconstriction or vein rupture [19]. Atasoy [19] suggested that tortuosity is not a problem for an interventionalist who has adequate US-guided technical skill. Following our experience, we recommend that all venous access be gained prior to injection of tumescence.

Tumescent fluid around the vein is as important in children as it is in adults undergoing endovenous laser ablation. The fluid serves three purposes. It compresses and reduces the diameter of the vein to be treated by apposing the vein wall closer to the laser fiber, thereby optimizing circumferential vein wall heating. Second, it acts as a “heat sink,” protecting adjacent tissues from thermal damage. Third, it can provide analgesia to the region being treated when local analgesia is added to the tumescent fluid [3]. In adults this is often a crystalloid fluid mixed with a local anesthetic agent, for example 500 mL saline, 25 mL 2% lidocaine and 10 mL sodium bicarbonate 8.4% [7]. In keeping with previous reports, [13] all the children in our cohort were treated under general anesthesia. When children are under general analgesia for the procedure, in keeping with the technique also reported by King et al. [13], we recommend that no local analgesia be added to the tumescent fluid.

As we encountered, there can be some technical problems in achieving and maintaining the tumescence for endovenous laser ablation. This can be problematic in patients with Klippel–Trenaunay syndrome when treating the embryonic marginal vein. However this vein is often surrounded by numerous small veins and does not have a surrounding fascial envelope. When fluid is injected around the vein there is often relatively quick dispersion of the fluid, which sometimes has to be reinjected to maintain adequate tumescence.

Compression post endovenous laser ablation for the treatment of varicose veins has been reported to reduce pain and improve function [20]; however there is little evidence upon which to base recommendations regarding the usage of compression stockings [21]. Although the use of compression post endovenous laser ablation has been reported in children, it was used for a short period of only 5 days post endovenous laser ablation [13]. In our cohort compression garments were worn by all our patients (using either 20–30 mmHg or 30–40 mmHg compression) for at least 6 weeks following endovenous laser ablation. We found that compliance in stocking wearing over the 6 weeks was good in all except two patients, both of whom required more than one endovenous laser ablation treatment to the same vein. Among children with venous diseases, compression garments can provide significant symptomatic relief for many patients and might prevent progressive expansion of venous lesions [22]. In toddlers there might be some practical challenges in obtaining a well-fitted compression stocking and therefore alternatives might be required. We recommend the temporary use of compression following endovenous laser ablation in children.

Complications of endovenous laser ablation are rare and include skin burns, nerve injury, arteriovenous fistula, endothermal heat-induced thrombosis and deep venous thrombosis [4]. Lower-extremity bruising and limb tightness are commonly reported after endovenous laser ablation, in 24% and 90% of patients, respectively [3]. No major complications have been reported in the few published pediatric cases to date [12, 13]. In our cohort, minor complications from endovenous laser ablation occurred in 2 of 35 patients (prolonged pain and a transient sensory nerve dysfunction). This gives a complications rate of 6% with a 95% confidence interval ranging 0–13%. Nerve injury can occur because of the proximity of nerves to ablated veins. Nerve injuries are reported in <1% of endovenous laser ablations [23]. The saphenous nerve is at risk of injury in the mid- to distal calf when the greater saphenous vein is ablated. The sural nerve is at greatest risk of injury in the distal calf where it is close to a treated short saphenous vein. Injury to these nerves results in cutaneous paresthesia, which is usually transient. The common peroneal nerve is a motor and sensory cutaneous nerve that descends within the lateral popliteal fossa, posterior to the head of the fibula, and is at greatest risk of injury in the region of the sapheno-popliteal junction [4]. Tumescence plays an important role in protection against nerve injury. Self-limiting local paresthesia lasting up to 3 weeks has been reported to have an incidence of 36.5% without tumescence [24].

In addition to endovenous laser ablation, there are numerous other methods to achieve vein closure. These include coil or vascular occlusion plug placement and adjuvant sclerotherapy. This study does not compare the outcomes of these various treatment modalities and a detailed discussion of these alternative techniques is beyond the scope of this report. It should, however, be noted that vessels are often too large for closure with standard coils and sometimes even vascular occlusion plugs; in addition, to perform effective sclerotherapy of such veins, larger volumes of sclerosant might be required than are permitted in small patients, risking impairment of renal function [25]. Occasionally, these methods might be required as an adjunct to endovenous laser ablation, as in 10 of our patients. When long veins need to be closed, such as the embryonic lateral marginal vein, the endovascular placement of sclerosant, coils or plugs might be needed to close the upper deep intrafacial segments of the vein before laser ablating the extrafascial component of the vein. Adequate tumescence is not possible in such deep locations; this increases the risk of failure of endovenous laser ablation and of damage to structures surrounding the vein. After the intrafascial segments of the vein are closed, endovenous laser ablation with tumescence can be safely undertaken to treat the extrafascial superficial segments of the vein. Another reason that adjunctive techniques are used to close veins was to minimize the risk of subsequent thromboembolism.

Although in this cohort endovenous laser ablation was found to be useful, there are some considerations regarding how generalizable the results are. The study was conducted at two large tertiary care institutions serving as regional referral centers for the management of children with vascular anomalies. As such, there is significant accumulated experience in the nuances of performing interventions in such patients. In addition, to undertake endovenous laser ablation, a disposable laser fiber and sheath and a laser source are required. A detailed cost analysis of the procedure is beyond the scope of the study. The disposable fiber and sheath kit costs approximately CAD $440. However the laser generator costs about CAD $30,000. Although laser ablation can be used for a variety of applications, such as osteoid osteoma ablation [26], the price of endovenous laser ablation might be considered high for low-volume institutions. For these reasons, the results presented here are generalizable only to institutions with a similar referral base and experience, whereas centers less used to managing patients with such pathologies might find the technique less useful.

This study has some other limitations that must be acknowledged. This is a retrospective review of a small cohort of children with limited patient numbers in each diagnostic category of venous disease. The duration of follow-up was limited and therefore conclusions regarding the durability of treatment cannot be made.

## Conclusion

Endovenous laser ablation expands the range of treatment options available for the management of a variety of venous anomalies in children. This study adds strength to previously published data confirming that endovenous laser ablation is feasible in children with an expected complication rate of 0–13%. In this cohort of patients, the aim of treatment was achieved in 83% of patients; however given the limitation of this and other studies of endovenous laser ablation in children, assessment of the technique in a larger number of children in whom there is longer-term follow-up is required.
